# A systematic review and meta‐analysis of the use of renin‐angiotensin system drugs and COVID‐19 clinical outcomes: What is the evidence so far?

**DOI:** 10.1002/prp2.666

**Published:** 2020-10-20

**Authors:** Amanj Kurdi, Nouf Abutheraa, Lina Akil, Brian Godman

**Affiliations:** ^1^ Strathclyde Institute of Pharmacy and Biomedical Sciences University of Strathclyde Glasgow UK; ^2^ Department of Pharmacology and Toxicology College of Pharmacy Hawler Medical University Kurdistan Region Government Erbil Iraq; ^3^ Division of Clinical Pharmacology Karolinska Institute Stockholm Sweden; ^4^ Division of Public Health Pharmacy and Management School of Pharmacy Sefako Makgatho Health Sciences University Pretoria South Africa

**Keywords:** angiotensin receptor blockers, angiotensin‐converting enzyme inhibitors, coronavirus, COVID‐19 infection, severe acute respiratory syndrome coronavirus 2

## Abstract

Conflicting evidence exists about the effect of angiotensin‐converting enzyme inhibitors (ACEIs)/angiotensin receptor blockers (ARBs) on COVID‐19 clinical outcomes. We aimed to provide a comprehensive/updated evaluation of the effect of ACEIs/ARBs on COVID‐19‐related clinical outcomes, including exploration of interclass differences between ACEIs and ARBs, using a systematic review/meta‐analysis approach conducted in Medline (OVID), Embase, Scopus, Cochrane library, and medRxiv from inception to 22 May 2020. English studies that evaluated the effect of ACEIs/ARBs among patients with COVID‐19 were included. Studies’ quality was appraised using the Newcastle‐Ottawa Scale. Data were analyzed using the random‐effects modeling stratified by exposure (ACEIs/ARBs, ACEIs, and ARBs). Heterogeneiity was assessed using I^2^ statistic. Several subgroup analyses were conducted to explore the impact of potential confounders. Overall, 27 studies were eligible. The pooled analyses showed nonsignificant associations between ACEIs/ARBs and death (OR:0.97, 95%CI:0.75,1.27), ICU admission (OR:1.09;95%CI:0.65,1.81), death/ICU admission (OR:0.67; 95%CI:0.52,0.86), risk of COVID‐19 infection (OR:1.01; 95%CI:0.93,1.10), severe infection (OR:0.78; 95%CI:0.53,1.15), and hospitalization (OR:1.15; 95%CI:0.81,1.65). However, the subgroup analyses indicated significant association between ACEIs/ARBs and hospitalization among USA studies (OR:1.59; 95%CI:1.03,2.44), peer‐reviewed (OR:1.93, 95%CI:1.38,2.71), good quality and studies which reported adjusted measure of effect (OR:1.30, 95%CI:1.10,1.50). Significant differences were found between ACEIs and ARBs with the latter being significantly associated with lower risk of acquiring COVID‐19 infection (OR:0.24; 95%CI: 0.17,0.34). In conclusion, high‐quality evidence exists for the effect of ACEIs/ARBs on some COVID‐19 clinical outcomes. For the first time, we provided evidence, albeit of low quality, on interclass differences between ACEIs and ARBs for some of the reported clinical outcomes.

**Abbreviations**
ACE2: angiotensin‐converting enzyme 2
ACEIs: angiotensin‐converting enzyme inhibitors
ARBs: angiotensin receptor blockers
AT_1_R: angiotensin receptor 1
CVD: cardiovascular disease

## 1 INTRODUCTION

Soon after the report of first clusters of COVID‐19 cases in China in December 2019, concerns were raised among clinicians and investigators that angiotensin‐converting enzyme inhibitors (ACEIs) and angiotensin receptor blockers (ARBs) might increase susceptibility to COVID‐19 infection and the likelihood of severe and fatal COVID‐19 illness.^1^ These concerns are based on the concept that angiotensin‐converting enzyme 2 (ACE2), an enzyme potentially upregulated by ACEIs/ARBs use, is the viral entry receptor that COVID‐19 uses to enter lung cell,^2^ coupled with the observation of high prevalence of hypertension and other cardiovascular comorbidities among COVID‐19 patients who have poor outcomes.^3^ Consequently, it was speculated that due to considerable prescription of ACEIs/ARBs to treat cardiovascular diseases (CVD), this would adversely affect outcomes from COVID‐19^4^ with underlying cardiac and kidney diseases already associated with poorer outcomes.^3^, ^5^, ^6^ Consequently, care to avoid treatments that well add to this.

Unsurprisingly, discussions regarding the potential impact of ACEIs/ ARBs have resulted in anxiety, which might cause patients and clinicians to discontinue or stop these medications.^7^ This should be avoided as there will be harm from the indiscriminate withdrawal of ACEIs/ARBs.^8^ This concern is complicated by uncertainty surrounding the upregulation of ACE2 by ACEIs/ARBs.^9^ Furthermore, the paradoxical protective role of ACEIs/ARBs in COVID‐19 patients is also being proposed.^10^ Due to these controversial findings, and despite consistent and reassuring recommendations for the continued use of ACEIs/ARBs in COVID‐19 patients issued by International Societies,^11^ these concerns remain. We wish to address this as we have already seen the impact that inappropriate endorsement of treatments can have on morbidity and mortality. Early endorsement of hydroxychloroquine resulted in drug shortages for other indications, price hikes, increased adverse drug reactions, and deaths from suicides.^12^, ^13^ However, subsequent studies failed to show clinical benefit resulting in the World Health Organisation (WHO) and the National Institute of Health (NIH) in the USA stopping the hydroxychloroquine arm in their studies.^14^, ^15^, ^16^ A similar situation has been seen with lopinavir/ritonavir.^15^ Consequently it is imperative that any considerations regarding management are evidence based.

We are aware that several observational studies have been conducted to address these concerns. However, these studies have reported conflicting findings which is a concern given the controversies with hydroxychloroquine and lopinavir/ritonavir. For instance, some studies^17^, ^18^, ^19^, ^20^, ^21^, ^22^ have reported a lower risk of severe COVID‐19 outcomes with ACEIs/ARBs while another study^23^ found a higher risk. Similarly, ACEIs/ARBs have been associated with lower mortality rates in some studies^17^, ^20^, ^24^, ^25^, ^26^, ^27^ while others^23^, ^28^ reported higher mortality rates. We are also aware that two recently published systematic reviews^29^, ^30^ containing 16 studies reported no evidence of any association between ACEIs/ARBs and mortality, severe COVID‐19 outcomes, or acquiring COVID‐19 infection; however, these studies only analyzed a limited range of outcomes, and did not report the effects of ACEIs and ARBs individually. The authors also did not undertake any subgroup analysis to explore the effect of potential confounders such as the study's quality and there are concerns that the findings may now be out‐dated. Furthermore, one of these studies^30^ only used narrative synthesis of the data. Consequently, we sought to undertake an updated and comprehensive evaluation of effect of ACEIs/ARBs use on all reported COVID‐19‐related outcomes, including exploration of any class differences, through a systematic review of the literature coupled with a meta‐analysis.

## 2 METHODS

### 2.1 Data source and searches

This systematic review and meta‐analysis was conducted and reported in accordance to the Preferred Reporting Items for Systematic Reviews and Meta‐Analyses (PRISMA) statement checklist.^32^ A protocol was drafted and shared with authors but not registered in any database as we did not want the submission of our findings to be delayed until the study protocol was registered as we wanted to provide the clinical community with a timely publication of the available evidence whether published in peer‐reviewed journals or awaiting publication surrounding the impact of ACEIs/ARBs use on COVID‐19 outcomes. The literature search was conducted in Embase, Medline (OVID), Scopus, Cochrane library, and medRxiv, from inception to 22 May 2020, using key terms related to ACEIs/ARBs and COVID‐19 concepts. A detailed electronic search strategy used in the database searches is attached [File S1]. We also manually searched the reference list of eligible articles to identify any further relevant articles.

### 2.2 Study selection

Eligibility criteria included original research studies, published in English, with COVID‐19 patients (target population) that reported the effects of ACEIs/ARBs (intervention), in comparison with non‐ACEIs/ARBs use (comparison), on COVID‐19‐related outcomes. No restrictions were placed on the reported outcomes or study types. All records identified from the search strategy were exported from the databases and imported into Covidence^®^ ^31^ whereby duplicate records were removed. Two reviewers (NA and LA) independently undertook titles and abstract screening for relevance, followed by selecting records for full‐text screening and data extraction. At each stage, discrepancies were resolved through discussion until consensus was achieved. A third author (AK) verified the eligibility of the included studies.

### 2.3 Data extraction and quality assessment

Data from the eligible studies were subsequently extracted by two authors (NA, AK) into a spreadsheet including information on the study characteristics (study design, setting, sample size, population, exposure‐ACEIs/ARBs, ACEIs, or ARBs) and outcome measures including death, intensive care unit (ICU) admission, risk of COVID‐19 infection, severe COVID‐19 infection, severe pneumonia, hospitalization, hospital discharge, use of ventilators, duration of hospital stay, septic shock, acute kidney injury, cardiac injury, and hospital readmission. Since the need for using ventilators typically necessitates ICU admission, we combined studies that reported ICU admission and ventilator use as a further composite outcome measure. Two authors (NA and LA) independently conducted the assessment of risk of bias using the Newcastle‐Ottawa Scale (NOS) for nonrandomized studies which consists of three domains (selection of participants and control (if applicable), comparability and exposure or outcome),^32^ whereby studies were classified into good quality (3 or 4 stars in selection domain AND 1 or 2 stars in comparability domain AND 2 or 3 stars in outcome domain), fair quality (2 stars in selection domain AND 1 or 2 stars in comparability domain AND 2 or 3 stars in outcome/exposure domain), and poor quality (0 or 1 star in selection domain OR 0 stars in comparability domain OR 0 or 1 stars in outcome/exposure domain)^33^; any disagreement between the two reviewers (NA and LA) was resolved by involving a third researcher (AK) for discussion until a consensus was reached. Furthermore, interrater reliability measures such as kappa statistic and percentage agreement were also calculated. Some of the coauthors have used this approach before.^34^

### 2.4 Data synthesis and analysis

For each study outcome that was reported by more than one study, the results from individual studies were combined statistically using the random‐effects meta‐analysis model, stratified by the level of exposure (ACEIs/ARBs, ACEIs, ARBs); whereas for outcomes which were reported by only one study, narrative synthesis was used. For studies which did not report the summary statistics and measure of effects, we firstly used the reported primary statistics (number of patients with/without the outcomes in both exposed/unexposed group) to calculate the corresponding measure of effects (Odds ratios‐ OR) and their 95% confidence interval (95%CI),^35^ and subsequently used these measure of effects in the random‐effects meta‐analysis; the random‐effects model was used as it is considered the most appropriate model by most researchers since it allows the results to be generalizable to other populations as well as addresses the likely heterogeneity between the included studies.^36^ Several subgroup analyses were also undertaken to explore the effect of potential confounders on the robustness and sensitivity of combined pooled estimates and included subgroup analyses based on whether the reported measure of effects was crude or adjusted, whether the study was peer‐reviewed or not, the study's methodological quality as per the risk of bias assessment was performed as well as the continent where the study was conducted. Meta‐analyses pooled estimates were presented as odds ratios and 95%CI and graphically as forest plots. Heterogeneity between the studies was evaluated using *I* ^2^ statistic,^37^ indicating whether variability is more likely due to study heterogeneity or chance. Negative *I* ^2^ values were set to zero, hence *I* ^2^ values ranged between 0% and 100% with 0% indicating lack of heterogeneity, whereas 25%, 50%, and 75% indicating low, moderate and high heterogeneity, respectively.^37^ Publication bias was assessed using funnel plots and Egger's asymmetry test^38^ for those outcomes where >10 studies were included in the analysis as recommended by Cochrane guidelines.^39^ Data were analyzed using STATA 12.

### 2.5 Role of the funding source

None.

## 3 RESULTS

### 3.1 Study characteristics

**FIGURE 1:**
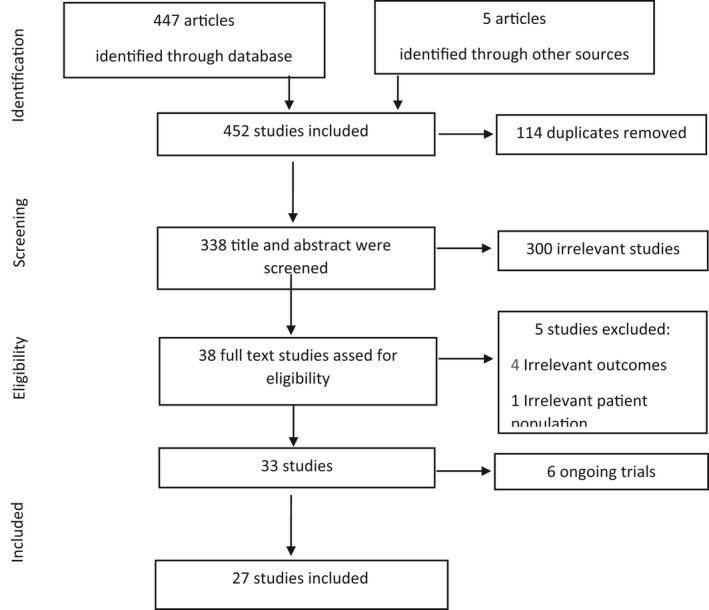
Study selection

The literature search identified 452 articles. However, only 27 studies were eligible for inclusion (Figure 1). A total of 72 372 patients were included in these 27 studies of which 10 197 (14.1%) patients were on ACEIs or ARBs. The average age of the population in these studies was 61 ± 9.6 years and men represented 52.24% of them (Table 1). Twenty‐one studies (77.8%) focused on comparing COVID‐19‐related outcomes between ACEI/ARB users vs nonusers among patients with COVID‐19 while the remaining six studies (22.2%) focused on comparing outcomes between ACEIs/ARBs users in patients with and without COVID‐19 infection (Table 1). ACEIs/ARBs in the included studies were indicated for a wide range of chronic conditions such as hypertension, coronary artery diseases, heart failure, diabetes, or chronic kidney disease.

**TABLE 1:** Study characteristics

	Population	Total n	Study Type	Exposure	n on RAAS inhibitors	Outcome(s)	Result (n or Odd Ratio + [95% confidence interval])
Bean et al (2020)^40^	All adult symptomatic inpatient testing positive for COVID‐19.	1200	Cohort	ACEIs/ARBs vs non‐ACEIs/ARBs among COVID‐19 patients	339	Death Critical care admission Death or critical care admission	n = 106/399 vs n = 182/801 n = 21/399 vs n = 106/801 0.63 (0.47‐0.84)
Benelli et al (2020)^41^	Patients tested positive for COVID‐19.	411	Cohort	ACEIs/ARBs vs non‐ACEIs/ARBs among COVID‐19 patients	110	Death ICU admission CPAP/NIV	n = 25/110 vs 47/301 n = 13/60 vs 15/301 n = 42/110 vs 70/301
Bravi et al (2020)^45^	Patients’ diagnosis of COVID‐19.	1603	Case‐control	ACEIs/ARBs vs non‐ACEIs/ARBs among COVID‐19 patients	450	Severe or very sever/lethal Very severe lethal	0.58 (0.34‐1.01) 0.87 (0.50‐1.49)
Chodick et al (2020)^49^	Patients with confirmed COVID‐19.	1317	Cohort	ACEIs/ARBs users in patients with and without COVID‐19	132	Increased risk for COVID‐19	1.19 (0.96‐1.47)
Dauchet et al (2020)^42^, *	Patients aged 35 years and over with suspected COVID‐19.	288	Cohort	ACEIs/ARBs vs non‐ACEIs/ARBs among COVID‐19 patients	109	COVID‐19+ Hospitalization ICU admission	Data reported for ACE inhibitor and ARBs separately
DeSpiegeleer et al (2020)^50^	All residents at two elderly care homes with confirmed COVID‐19.	154	Cohort	ACEIs/ARBs vs non‐ACEIs/ARBs among COVID‐19 patients	30	Serious COVID‐19	0.48 (0.10‐1.97)
Feng et al (2020)^19^	Patients diagnosed with COVID‐19.	467	Cohort	ACEIs/ARBs vs non‐ACEIs/ARBs among COVID‐19 patients	33	Disease severity: Moderate Severe Critical	n = 29/33 vs 319/443 n = 2/33 vs 52/443 n = 2/33 vs 68/443
Feng et al (2020)^51^	All adult patients with confirmed COVID‐19.	564	Cohort	ACEIs/ARBs vs non‐ACEIs/ARBs among COVID‐19 patients	16	Disease severity	0.41 (0.05‐3.19)
Guo et al (2020)^28^	Patients with COVID‐19	187	Cohort	ACEIs/ARBs vs non‐ACEIs/ARBs among COVID‐19 patients	19	Death	n = 7/ 19 vs n = 36/168
Ip Andrew et al (2020)^27^	Patients hospitalized with confirmed COVID‐19	3017	Cohort	ACEIs/ARBs vs non‐ACEIs/ARBs among COVID‐19 patients	NR	Death (expired) Discharged	1.6 [1.23‐1.99] n = 323 vs 407
Khawaja et al (2020)^52^	Patients hospitalized with COVID −19	605	Cohort	ACEIs/ARBs users in patients with and without COVID‐19	125	Hospitalization with COVID‐19	Data reported for ACE inhibitor and ARBs separately
Khera et al (2020)^46^	Patients receiving antihypertensive agents and tested positive for COVID‐19.	2263	Cohort	ACEIs/ARBs vs non‐ACEIs/ARBs among COVID‐19 patients	852	Hospitalization Mortality	Data reported for ACE inhibitor and ARBs separately
Li et al (2020)^24^	Patients with COVID‐19 and hypertension	1178	Cohort	ACEIs/ARBs vs non‐ACEIs/ARBs among COVID‐19 patients	115	Severity Death	n = 57/115 vs 116/247 n = 21/115 vs 56/247
Liu et al (2020)^18^	All patients were diagnosed with COVID‐19 and hypertension	78	Cohort	ACEIs/ARBs vs non‐ACEIs/ARBs among COVID‐19 patients	12	Disease severity	Data reported for ACE inhibitor and ARBs separately
Mancia et al (2020)^21^	Patients 40 years of age or older with a Positive test of COVID −19	6272	Case‐control	ACEIs/ARBs users in patients with and without COVID‐19	2896	Critical or fatal of clinical manifestations	Data reported for ACE inhibitor and ARBs separately
Mehta et al (2020)^44^	Patients tested for COVID‐19 and had ACEI or ARB prescribed.	18 472	Cohort	ACEIs/ARBs vs non‐ACEIs/ARBs among COVID‐19 patients	212	COVID‐19+ Hospital admission ICU admission Use of ventilator	0.97[0.81‐1.15] 1.93 (1.38‐2.71) 1.64 (1.07‐2.51) 1.32 (0.80‐2.18)
Meng et al (2020)^17^	Patients with positive COVID‐19.	42	Cohort	ACEIs/ARBs vs non‐ACEIs/ARBs among COVID‐19 patients	17	Hospitalization Hospital discharge Severity of disease Death	4 days vs 2 days 20 days vs 16.5 days OR:0.33[0.09‐1.31] n = 0/17 vs n = 1/25
Raisi‐Estabragh et al (2020)^53^	Individuals tested for COVID‐19 aged 40‐69 years old.	1474	Cohort	ACEIs/ARBs users in patients with and without COVID‐19	312	COVID+	0.956[0.695‐1.316]
Rentsch et al (2020)^43^	Veterans aged 54‐75 years with positive COVID‐19 test	585	Cohort	ACEIs/ARBs vs non‐ACEIs/ARBs among COVID‐19 patients	255	COVID‐19+ Hospitalization ICU admission	0.93[0.78‐1.23] 1.24[0.79‐1.95] 1.69[1.01‐2.84]
Reynolds et al (2020)^22^	Patients who were tested for COVID‐19.	12 594	Cohort	ACEIs/ARBs vs non‐ACEIs/ARBs among COVID‐19 patients	2319	COVID‐19+ Severity of COVID‐19	1110/1909 vs 1101/1909 275/1110 vs 274/1101
Rhee et al (2020)^54^	Patients with confirmed COVID‐19	832	Cohort	ACEIs/ARBs vs non‐ACEIs/ARBs among COVID‐19 patients	327	ICU admission or death	0.599[0.251‐1.431]
Richardson et al (2020)^23^	All patients who were hospitalized with COVID‐19 infection.	5700	Cohort	ACEIs/ARBs vs non‐ACEIs/ARBs among COVID‐19 patients	413	Invasive mechanical ventilation ICU care Readmission Discharged home Death	n = 79/413 vs n = 122/953 n = 87/413 vs 141/953 n = 6/413 vs n = 18/953 n = 261/413 vs 639/953 n = 130/413 vs 254/953
Rossi et al (2020)^47^	All symptomatic patients who tested positive for COVID‐19.	2653	Cohort	ACEIs/ARBs vs non‐ACEIs/ARBs among COVID‐19 patients	450	Death Hospitalization	0.8[0.50‐1.3] 1.12 [0.82‐1.54]
Yan et al (2020)^48^	Patients with confirmed diagnosis of COVID −19 infection.	610	Case‐control	ACEIs/ARBs users in patients with and without COVID‐19	NR	COVID‐19+ Disease severity of COVID‐19 severe + critical vs mild + common	Data reported for ACE inhibitor and ARBs separately
Yang et al (2020)^25^	Patients with confirmed COVID‐19.	462	Cohort	ACEIs/ARBs vs non‐ACEIs/ARBs among COVID‐19 patients	43	Tested positive for COVID‐19 Days patient remained in hospital (mean ± SD) Critical severity Death	n = 43 vs n = 83 35.2 ± 12.8 vs 37.5 ± 12.3. n = 4 vs n = 19 n = 2 vs n = 11
Zeng et al (2020)^26^	Adult patients with suspected and confirmed cases of COVID‐19.	274	Cohort	ACEIs/ARBs vs non‐ACEIs/ARBs among COVID‐19 patients	28	Mortality length of hospital stays (days) discharge rate hospitalization rate. Tested positive for COVID Severe pneumonia	n = 2/28 vs n = 5/47 n = 21(15.25) vs n = 22 (16‐28) n = 21/28 vs, n = 29/47 n = 5/28 vs n = 13/47 n = 20/28 vs n = 31/47 n = 15/28 vs n = 15/47
Zhang et al (2020)^20^	Patients diagnosed with COVID‐19,	1128	Cohort	ACEIs/ARBs vs non‐ACEIs/ARBs among COVID‐19 patients	188	Mortality Acute respiratory distress syndrome Septic shock Acute kidney injury Cardiac injury	0.37 [0.15‐0.89] 0.65 [0.41‐1.04] 0.32 [0.13‐0.80] 0.78 [0.37‐1.65] 0.78 [0.44‐1.32]

ACEIs, Angiotensin‐converting‐enzyme inhibitors; ARBs, Angiotensin II receptor blockers; COVID, coronavirus disease; CPAP, continuous positive airway pressure; ICU, intensive care unit; n, number of patients; NIV, noninvasive ventilation; NR, not reported; OR, odds ratio; RAAS, Renin‐Angiotensin‐Aldosterone System; SD, standard deviation.

* This study reported data from two cohorts; hence it is included twice in the analyses.

In terms of outcomes, nine studies (33.3%) reported three to five COVID‐19‐related outcomes,^20^, ^23^, ^25^, ^26^, ^40^, ^41^, ^42^, ^43^, ^44^ while another nine studies (33.3%) reported only two outcomes^17^, ^19^, ^22^, ^24^, ^27^, ^45^, ^46^, ^47^, ^48^ with another one‐third reported only one outcome.^19^, ^22^, ^29^, ^46^, ^47^, ^48^, ^49^, ^50^, ^51^ Overall, the 27 studies reported data on 15 unique outcomes including death in 12 studies,^18^, ^21^, ^28^, ^49^, ^50^, ^51^, ^52^, ^53^, ^54^ ICU admission in seven studies,^23^, ^25^, ^40^, ^41^, ^42^, ^43^, ^44^ death/ICU admission as a composite outcome in four studies,^21^, ^40^, ^45^, ^54^ risk of acquiring COVID‐19 infection in nine studies,^22^, ^25^, ^26^, ^42^, ^43^, ^44^, ^48^, ^49^, ^53^ risk of severe COVID‐19 infection in seven studies,^17^, ^18^, ^19^, ^22^, ^24^, ^48^, ^50^ risk of severe pneumonia in two studies,^26^, ^51^ risk of hospitalization in eight studies,^26^, ^42^, ^43^, ^44^, ^45^, ^46^, ^47^, ^52^ hospital discharge in three studies,^23^, ^26^, ^27^ use of ventilator in four studies,^19^, ^23^, ^41^, ^44^ duration of hospital stay in two studies,^25^, ^26^ and each of acute respiratory distress syndrome (ARDS), septic shock, cardiac shock, acute kidney injury,^20^ and hospital readmission^23^ in one study, respectively. In terms of the exposure, the effects of ACEIs and ARBs were assessed as one class (ACEIs/ARBs) in 17 studies (63%),^17^, ^40^, ^43^, ^44^, ^47^, ^50^, ^51^, ^53^, ^54^ as separate classes in five studies (18.5%) 52, 74, 78, 80, 84), and both as one and separate classes in another five studies.^18^, ^19^, ^41^, ^45^, ^49^

The majority of the 27 eligible studies were conducted in Asia (44.4%, n = 12 with 10 studies from China, one each from Korea and Israel), followed by nine studies (33.3%) from Europe (four in Italy, three in the United Kingdom and one each from France and Belgium) and the remaining six (22.3%) from the USA. Furthermore, the reported measure of effects were crude/unadjusted measures in the majority of the studies (77.8%, n = 21)^18^, ^19^, ^40^, ^41^, ^42^, ^43^, ^44^, ^45^, ^46^, ^48^, ^53^, ^54^; with most of them (59.3%, n = 16) being nonpeer‐reviewed articles published as preprints on medRivix,^24^, ^50^, ^51^, ^52^, ^53^, ^54^ and only four rated as a good quality studies based on the Newcastle‐Ottawa Quality Assessment risk of bias^21^, ^40^, ^47^, ^48^ (Table 2). Results from the interrater reliability measures indicated a substantial agreement between the two independent reviewers (NA and LA) in assessing the risk of bias (kappa statistic = 0.79; percentage of agreement = 89% (24/27)).

**TABLE 2:** Quality assessment score of the studies included into the systematic review and meta‐analysis based on the using the Newcastle‐Ottawa Scale

Cohort studies													
N	Author (Month, year)	Selection				Comparability		Outcome			Final score		Score Quality**
1	Bean et al (2020)^40^	B*	C	A*	A*	Demographic*	Comorbidities*	B*	A*	C	7		Good
2	Benelli et al (2020)^41^	B*	C	A*	A*	—	—	B*	No	C	4		Poor
3	Chodick et al (2020)^49^	B*	C	A*	A*	Demographic*	Comorbidities*	B*	NA	D	6		Poor
4	DeSpiegeleer et al (2020)^50^	B*	C	A*	A*	Demographic*	Comorbidities*	B*	NA	D	6		Poor
5	Feng et al (2020)^19^	B*	C	A*	A*	—	—	B*	NA	D	4		Poor
6	Feng et al (2020)^51^	B*	C	A*	A*	—	—	B*	NA	D	4		Poor
7	Khawaja et al (2020)^52^	A*	A*	A*	A*	Demographic*	Comorbidities*	B*	NA	D	7		Poor
8	Khera et al (2020)^46^	B*	A*	A*	A*	—	—	B*	NA	D	5		Poor
9	Li et al (2020)^24^	B*	C	A*	A*	—	—	B*	NA	D	4		Poor
10	Dauchet et al (2020)^42^	B*	A*	A*	A*	—	—	B*	NA	D	5		Poor
11	Ip Andrew et al (2020)^27^	B*	C	A*	A*	—	—	B*	NA	D	4		Poor
12	Liu et al (2020)^18^	A*	C	A*	A*	—	—	B*	NA	D	4		Poor
13	Mehta et al (2020)^44^	A*	A*	A*	A*	—	—	B*	NA	D	5		Poor
14	Raisi‐Estabragh et al (2020)^53^	B*	A*	A*	A*	—	—	B*			5		Poor
15	Rhee et al (2020)^54^	A*	A*	A*	A*	Demographic*	Comorbidities*	B*	NA	D	7		Poor
16	Yang et al (2020)^25^	B*	A*	A*	A*	—	—	B*	B	D	5		Poor
17	Zeng Zh et al (2020)^26^	B*	A*	A*	A*	—	—	B*	A*	A*	7		Poor
18	Zhang et al (2020)^20^	A*	A*	A*	A*	Demographic*	Comorbidities*	B*	NA	D	7		Poor
19	Rossi et al (2020)^47^	A*	C	A*	A*	Demographic*	Comorbidities*	B*	A*	A*	8		Good
20	Reynolds et al (2020)^22^	B*	A*	A*	A*	Demographic*	Comorbidities*	B*	NA	D	7		Poor
21	Rentsch et al (2020)^43^	B*	C	A*	A*	—	—	B*	NA	D	4		Poor
22	Meng et al (2020)^17^	B*	C	A*	A*	—	—	B*	NA	D	4		Poor
23	Guo et al (2020)^28^	A*	C	A*	A*	—	—	B*	NA	D	4		Poor
24	Richardson et al (2020)^23^	A*	C	A*	A*	—	—	B*	B	D	4		Poor
Case‐control studies													
25	Bravi et al (2020)^45^	A*	A*	A*	A*	—	—	A*	A*	C	6	Poor	
26	Mancia et al (2020)^21^	A*	A*	A*	A*	—	Comorbidities *	A*	A*	C	7	Good	
27	Yan et al (2020)^48^	A*	A*	A*	A*	Demographic*	—	B*	A*	D	6	Good	

** Studies were classified into good quality (3 or 4 stars in selection domain AND 1 or 2 stars in comparability domain AND 2 or 3 stars in outcome domain), fair quality (2 stars in selection domain AND 1 or 2 stars in comparability domain AND 2 or 3 stars in outcome/exposure domain) and poor quality (0 or 1 star in selection domain OR 0 stars in comparability domain OR 0 or 1 stars in outcome/exposure domain) (33).

### 3.2 Study outcomes

#### 3.2.1 Death and ICU admission

**FIGURE 2:**
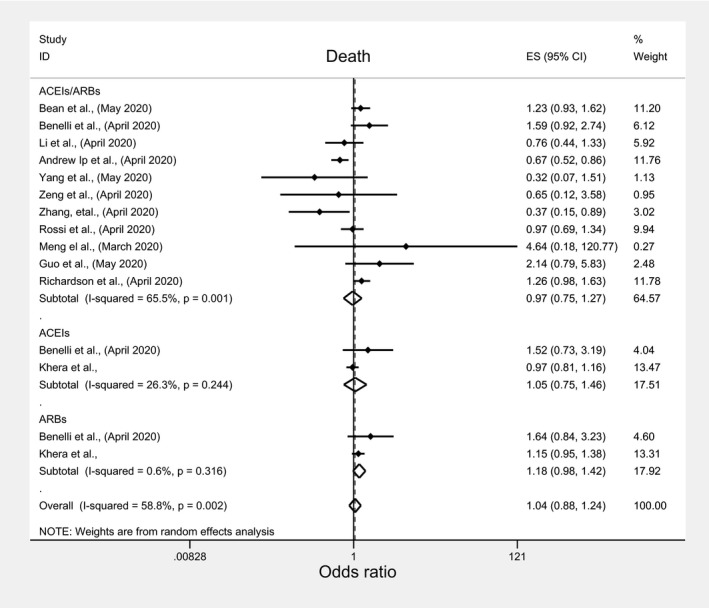
Forest plot depicting pooled estimates for the association between mortality and the three levels of renin‐angiotensin system drug exposure (ACEIs/ARBs, ACEIs, ARBs)

**FIGURE 3:**
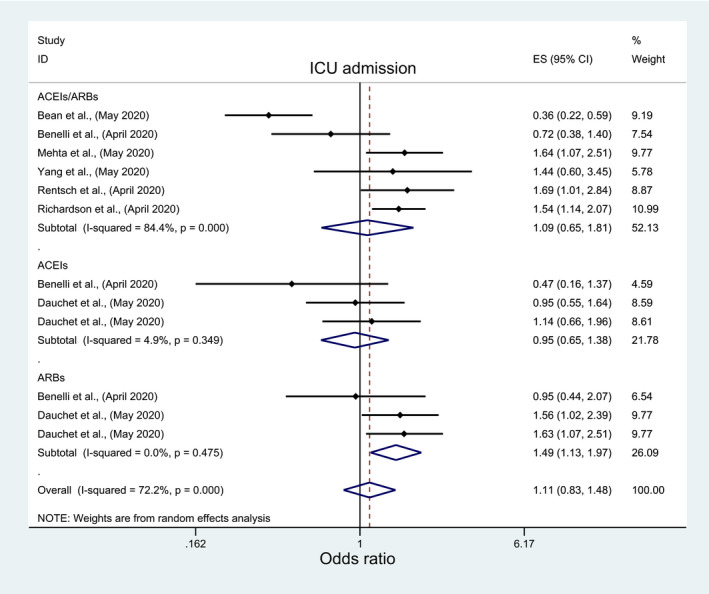
Forest plot depicting pooled estimates for the association between Intensive Care Unit admission and the three levels of renin‐angiotensin system drug exposure (ACEIs/ARBs, ACEIs, ARBs)

**FIGURE 4:**
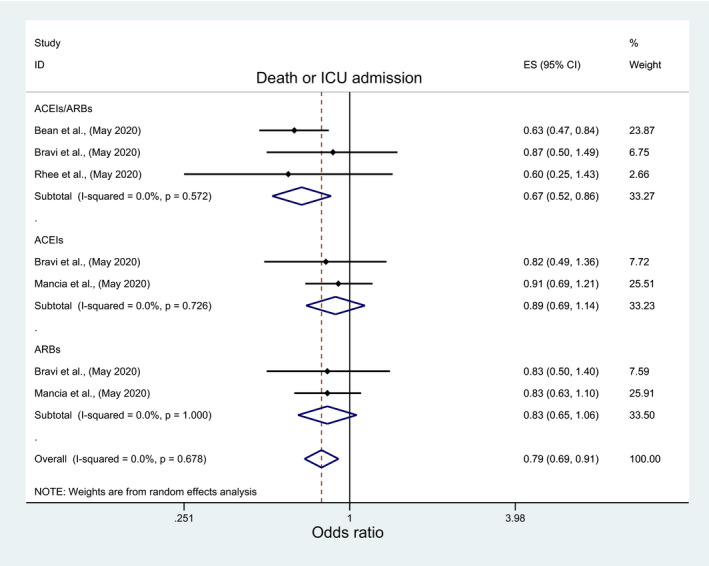
Forest plot depicting pooled estimates for the association between the composite outcome of mortality/ Intensive Care admission and the three levels of renin‐angiotensin system drug exposure (ACEIs/ARBs, ACEIs, ARBs)

Among pertinent studies, there was insignificant association between mortality and ACEIs/ARBs (OR: 0.97; 95%CI: 0.75 1.27), ACEIs (OR:1.05; 95%CI: 0.75, 1.46), or ARBs (OR:1.18, 95%CI: 0.98, 1.42) (Figure 2; Table 3), regardless of the studies’ country, quality, peer‐review status or crude/adjusted measure of effect (File S2; Table 4). Similarly, there was an insignificant association between ICU admission and ACEIs/ARBs (OR: 1.09; 95%: 0.65, 1.81) and ACEIs (OR:0.95; 95%CI: 0.65, 1.38) but significantly higher odds of ICU admission with ARBs (OR:1.49, 95%CI: 1.13, 1.97) (Figure 3; Table 3). However, subgroup analyses indicated different results. A significantly lower ICU admission rate was associated with ACEIs/ARBs among European studies (OR:0.49; 95%CI: 0.25, 0.97), and good quality studies (OR:0.36; 95%CI: 0.22, 0.59), in contrast to significantly higher ICU admission rate among USA studies (OR:1.59; 95%CI: 1.28, 1.98), peer‐reviewed studies (OR:1.56; 95%CI: 1.23, 1.97), and poor quality studies (OR:1.44; 95%CI: 1.13, 1.84) (File S3; Table 4). Meta‐analysis of the three studies that reported death and ICU admission as a composite endpoint indicated significantly lower odds of death/ICU admission with ACEIs/ARBs use (OR:0.67; 95%CI: 0.52, 0.86) but insignificant lower association with ACEIs (OR:0.89; 95%CI: 0.69, 1.14) or ARBs (OR: 0.83; 95%CI: 0.65, 1.06), regardless of any subgroup analysis for ACEIs and ARBs (Figure 4; Table 3). The subgroup analyses for ACEIs/ARBs, however, showed a significantly lower association of death/ICU admission with ACEIs/ARBs only among European studies (OR: 0.68; 95%CI: 0.52, 0.89), good quality studies (OR:0.63; 95%CI: 0.47, 0.84), and studies which reported adjusted measure of effect (OR:0.63; 95%CI: 0.47, 0.84) (File S4; Table 4).

**TABLE 3:** Meta‐analyses pooled estimates with 95%CI of the effects of ACEIs/ARBs on COVID‐19 related clinical outcomes

Outcomes	ACEIs/ARBs	*P*‐value	ACEIs	*P*‐value	ARBs	*P*‐value
Death	0.973 (0.746, 1.269)	0.84	1.049 (0.751, 1.464)	0.781	1.181 (0.983, 1.418)	0.076
Number of studies	11		2		2	
I‐squared	65.5%	0.001	26.3%	0.244	0.6%	0.316
ICU	1.086 (0.652, 1.809)	0.75	0.945 (0.65, 1.376)	0.769	1.49 (1.126, 1.973)	0.005
Number of studies	6		3		3	
I‐squared (*P*‐value)	84.4%	<0.001	4.9%	0.349	0%	0.475
Death/ICU	0.67 (0.524, 0.857)	0.001	0.888 (0.694, 1.136)	0.345	0.83 (0.65, 1.061)	0.136
Number of studies	3		2		2	
I‐squared (*P*‐value)	0%	0.572	0%	0.726	0%	1.000
Risk of COVID‐19	1.014 (0.935, 1.099)	0.745	1.133 (1.417, 21.27)	0.273	0.557 (0.107, 2.895)	0.46
Number of studies	7		3		2	
I‐squared (*P*‐value)	0%	0.75	0%	0.457	97.9%	<0.001
Severe COVID‐19	0.782 (0.529, 1.154)	0.215	0.718 (0.264, 1.955)	0.517	0.506 (0.247, 1.036)	0.062
Number of studies	6		3		3	
I‐squared (*P*‐value)	43.3%	0.117	0%	0.799	18%	0.296
Severe pneumonia	1.285 (0.237, 6.958)	0.771	NA		NA	
Number of studies	2					
I‐squared (*P*‐value)	57.5%	0.125				
Hospitalization	1.153 (0.806, 1.65)	0.436	1.077 (0.791, 1.465)	0.638	0.907 (0.74, 1.112)	0.349
Number of studies	5		5		5	
I‐squared (*P*‐value)	74.5%	0.003	63.7%	0.026	0%	0.965
Hospital discharge	1.213 (0.739, 1.991)	0.446	NA		NA	
Number of studies	3					
I‐squared (*P*‐value)	82.2%	0.004				
Ventilator use	1.492 (0.804, 2.77)	0.205	1.014 (0.03, 34.758)	0.994	0.985 (0.084, 11.57)	0.990
Number of studies	4		2		2	
I‐squared (*P*‐value)	80.7%	0.001	64.7%	0.092	88.6%	0.003
ICU/ventilator use	1.225 (0.836, 1.795)	0.298	1.149 (0.554, 2.382)	0.709	1.467 (0.907, 2.373)	0.118
Number of studies	10		5		5	
I‐squared (*P*‐value)	83.2%	<0.001	75.2%	0.003	66.2%	<0.001

Note

NA, not applicable indicating no enough studies to perform meta‐analyses

**TABLE 4:** Subgroup meta‐analyses pooled estimates with 95%CI of the effects of ACEIs/ARBs on COVID‐19 related clinical outcomes

	Death (n = 15)		
	ACEIs/ARBs	ACEIs	ARBs
Adjusted outcome measure			
Adjusted OR	0.973 (0.260, 1.660)	NA	NA
Crude OR	1.048 (0.772, 1.424)	1.049 (0.751, 1.464)*	1.181 (0.983, 1.418)*
Number of studies	2 vs 9	0 vs 2	0 vs 2
Peer‐reviewed article?			
Yes	0.894 (0.522, 1.533)	NA	NA
No	1.004 (0.716, 1.408)	1.049 (0.751, 1.464)*	1.181 (0.983, 1.418)*
Number of studies	6 vs 5	0 vs 2	0 vs 2
Study's quality			
Good quality	1.113 (0.884, 1.400)	NA	NA
Poor quality	0.915 (0.627, 1.336)	1.049 (0.751,1.464)*	1.181 (0.983,1.418)*
Number of studies	2 vs 9	0 vs 2	0 vs 2
Study's country			
Europe	1.176 (0.932, 1.483)	1.523 (0.728, 3.185)	1.645 (0.838, 3.229)
USA	0.92 (0.494, 1.714)	0.97 (0.811, 1.161)	1.15 (0.954, 1.386)
Asia	0.753 (0.401, 1.413)	NA	NA
Number of studies	3 vs 2 vs 6	1 vs 1 vs 0	1 vs 1 vs 0

	ICU admission (n = 12)		
Adjusted outcome measure			
Adjusted OR	NA	NA	NA
Crude OR	1.086 (0.652, 1.809)*	0.945 (0.650, 1.376)*	1.490 (1.126, 1.973)*
Number of studies	0 vs 6	0 vs 3	0 vs 3
Peer‐reviewed article?			
Yes	1.560 (1.234, 1.972)	NA	NA
No	0.762 (0.295, 1.972)	0.945 (0.650, 1.376)*	1.490 (1.126, 1.973)*
Number of studies	3 vs 3	0 vs 3	0 vs 3
Study's quality			
Good quality	0.364 (0.224, 0.591)	NA	NA
Poor quality	1.445 (0.133, 1.843)	0.945 (0.650, 1.376)*	1.490 (1.126, 1.973)*
Number of studies	1 vs 5	0 vs 3	0 vs 3
Study's country			
Europe	0.495 (0.253, 0.966)	0.945 (0.650, 1.376)*	1.490 (1.126, 1.973)*
USA	1.591 (1.277, 1.983)	NA	NA
Asia	1.439 (0.600, 3.453)	NA	NA
Number of studies	2 vs 3. vs 1	3 vs 0. vs 0	3 vs 0. vs 0

	Death/ICU admission (n = 7)		
Adjusted outcome measure			
Adjusted OR	0.630 (0.471, 0.842)	NA	NA
Crude OR	0.783 (0.493, 1.243)	0.888 (0.694, 1.136)*	0.830 (0.650, 1.061)*
Number of studies	1 vs 2	0 vs 2	0 vs 2
Peer‐reviewed article?			
Yes	NA	0.910 (0.690, 1.210)	0.830 (0.630, 1.100)
No	0.670 (0.524, 0.857)*	0.820 (0.490, 1.360)	0.830 (0.500, 1.400)
Number of studies	0 vs 3	1 vs 1	1 vs 1
Study's quality			
Good quality	0.630 (0.471, 0.842)	0.910 (0.687, 1.205)	0.830 (0.628, 1.097)
Poor quality	0.783 (0.493, 1.243)	0.820 (0.492, 1.366)	0.830 (0.496, 1.389)
Number of studies	1 vs 2	1 vs 1	1 vs 1
Study's country			
Europe	0.679 (0.520, 0.887)	0.888 (0.694, 1.136)	0.830 (0.650, 1.061)
USA	NA	NA	NA
Asia	0.599 (0.251, 1.430)	NA	NA
Number of studies	2 vs 0 vs 1	2 vs 0 vs 0	2 vs 0 vs 0

	Risk of COVID‐19 infection (n = 12)		
Adjusted outcome measure			
Adjusted OR	1.190 (0.962, 1.473)	1.180 (0.867, 1.605)	1.290 (0.930, 1.790)
Crude OR	0.986 (0.904, 1.077)	1.015 (0.620, 1.662)	0.240 (0.170, 0.340)
Number of studies	1 vs 6	1 vs 2	1 vs 1
Peer‐reviewed article?			
Yes	1.030 (0.941, 1.128)	1.180 (0.867, 1.605)	1.290 (0.930, 1.790)
No	0.948 (0.790, 1.138)	1.015 (0.620, 1.662)	0.240 (0.170, 0.340)
Number of studies	4 vs 3	1 vs 2	1 vs 1
Study's quality			
Good quality	NA	0.650 (0.265, 1.597)	0.240 (0.170, 0.339)
Poor quality	1.014 (0.935, 1.099)*	1.176 (0.933, 1.481)	1.290 (0.930, 1.790)
Number of studies	0 vs 7	1 vs 2	1 vs 1
Study's country			
Europe	0.956 (0.695, 1.316)	1.170 (0.825, 1.660)	NA
USA	0.99 (0.901, 1.087)	NA	NA
Asia	1.131 (0.942, 1.358)	1.023 (0.622, 1.684)	0.557 (0.107, 2.895)*
Number of studies	1 vs 3 vs 3	1 vs 0 vs 2	0 vs 0 vs 2

	Severe COVID‐19 (n = 12)		
Adjusted outcome measure			
Adjusted OR	0.480 (0.108, 2.130)	NA	NA
Crude OR	0.795 (0.525, 1.206)	0.718 (0.264, 1.955)*	0.506 (0.247, 1.036)*
Number of studies	1 vs 5	0 vs 3	0 vs 3
Peer‐reviewed article?			
Yes	0.895 (0.614, 1.303)	0.595 (0.067, 5.296)	0.333 (0.069, 1.607)
No	0.387 (0.144, 1.040)	0.755 (0.245, 2.328)	0.509 (0.176, 1.474)
Number of studies	4 vs 2	1 vs 2	1 vs 2
Study's quality			
Good quality	NA	1.230 (0.190, 7.946)	0.770 (0.362, 1.638)
Poor quality	0.782 (0.529, 1.154)*	0.578 (0.176, 1.893)	0.283 (0.101, 0.792)
Number of studies	0 vs 6	1 vs 2	1 vs 2
Study's country			
Europe	0.480 (0.108, 1.130)	NA	NA
USA	0.994 (0.820, 1.205)	NA	NA
Asia	0.513 (0.216, 1.216)	0.718 (0.264, 1.955)*	0.506 (0.247, 1.036)*
Number of studies	1 vs 1 vs 4	0 vs 0 vs 3	0 vs 0 vs 3

	Severe pneumonia (n = 2)		
Adjusted outcome measure			
Adjusted OR	0.410 (0.050, 3.275)	NA	NA
Crude OR	2.462 (0.939, 6.452)	NA	NA
Number of studies	1 vs 1		
Peer‐reviewed article?			
Yes	NA	NA	NA
No	1.285 (0.237, 6.958)	NA	NA
Number of studies	0 vs 2		
Study's quality			
Good quality	NA	NA	NA
Poor quality	1.285 (0.237, 6.958)	NA	NA
Number of studies	0 vs 2		
Study's country			
Europe	NA	NA	NA
USA	NA	NA	NA
Asia	1.285 (0.237, 6.958)		
Number of studies	0 vs 0 vs 2		

	Hospitalization (n = 15)		
Adjusted outcome measure			
Adjusted OR	1.300 (1.113, 1.518)	1.170 (0.900, 1.520)	1.0 (0.702, 1.424)
Crude OR	1.032 (0.561, 1.897)	1.056 (0.684, 1.631)	0.865 (0.674, 1.109)
Number of studies	1 vs 4	1 vs 4	1 vs 4
Peer‐reviewed article?			
Yes	1.930 (1.377, 2.705)	NA	NA
No	0.977 (0.647, 1.474)	1.077 (0.791, 1.465)*	0.907 (0.740, 1.112)*
Number of studies	1 vs 4	0 vs 5	0 vs 5
Study's quality			
Good quality	1.300 (1.113, 1.518)	NA	NA
Poor quality	1.032 (0.561, 1.897)	1.077 (0.791, 1.465)*	0.907 (0.740, 1.112)*
Number of studies	1 vs 4	0 vs 5	0 vs 5
Study's country			
Europe	0.907 (0.413, 1.992)	1.181 (0.843, 1.656)	0.922 (0.721, 1.179)
USA	1.589 (1.033, 2.443)	0.77 (0.527, 1.124)	0.877 (0.611, 1.258)
Asia	0.569 (0.178, 1.815)	NA	NA
Number of studies	2 vs 2 vs 1	4 vs 1 vs 0	4 vs 1 vs 0

	Hospital discharge (n = 3)		
Adjusted outcome measure			
Adjusted OR	NA	NA	NA
Crude OR	1.213 (0.739, 1.991)	NA	NA
Number of studies	0 vs 3		
Peer‐reviewed article?			
Yes	0.844 (0.663, 1.074)	NA	NA
No	1.513 (1.184, 1.935)	NA	NA
Number of studies	1 vs 2		
Study's quality			
Good quality	NA	NA	NA
Poor quality	1.213 (0.739, 1.991)	NA	NA
Number of studies	0 vs 3		
Study's country			
Europe	NA	NA	NA
USA	1.122 (0.641, 1.964)	NA	NA
Asia	1.862 (0.659, 5.26)	NA	NA
Number of studies	0 vs 2 vs 1		

	Ventilator use (n = 8)		
Adjusted outcome measure			
Adjusted OR	NA	NA	NA
Crude OR	1.492 (0.804, 2.770)	1.014 (0.03, 34.758)	0.985 (0.084, 11.57)
Number of studies	0 vs 4	0 vs 2	0 vs 2
Peer‐reviewed article?			
Yes	1.141 (0.606, 2.150)	0.078 (0.001, 6.878)	0.251 (0.053, 1.185)
No	3.338 (2.035, 5.475)	3.603 (1.889, 6.872)	3.129 (1.699, 5.761)
Number of studies	1 vs 3	1 vs 1	1 vs 1
Study's quality			
Good quality	NA	NA	NA
Poor quality	1.492 (0.804, 2.770)	1.014 (0.030, 34.758)	0.985 (0.084, 11.570)
Number of studies	0 vs 4	0 vs 2	0 vs 2
Study's country			
Europe	3.338 (2.035, 5.475)	3.603 (1.889, 6.872)	3.129 (1.699, 5.762)
USA	1.524 (1.171, 1.985)	NA	NA
Asia	0.202 (0.043, 0.947)	0.078 (0.001, 6.469)	0.251 (0.053, 1.187)
Number of studies	1 vs 2 vs 1	1 vs 0 vs 1	1 vs 0 vs 1

* Indicates that the pooled estimate is the same as the overall analyses because all the studies were in one group; NA: not applicable indicating that no studies were available to perform meta‐analyses for these outcomes;

#### 3.2.2 Risk of acquiring COVID‐19 infection, severe COVID‐19 infection and severe pneumonia

**FIGURE 5:**
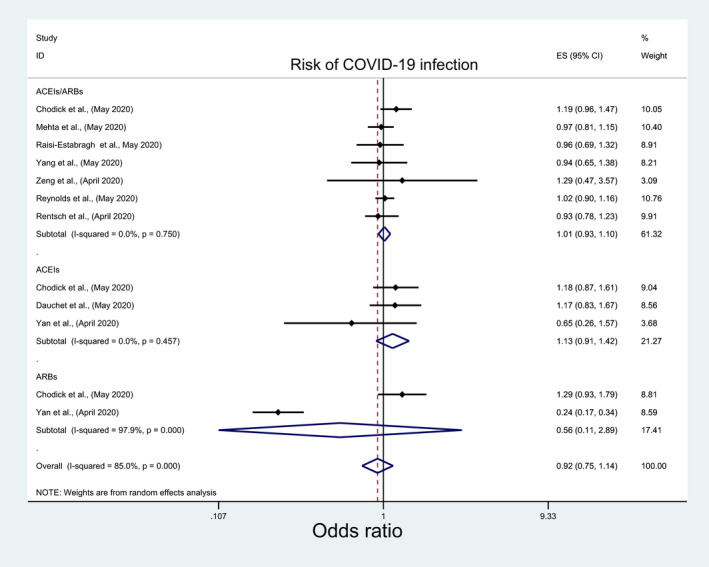
Forest plot depicting pooled estimates for the association between risk of acquiring COVID‐19 infection and the three levels of renin‐angiotensin system drug exposure (ACEIs/ARBs, ACEIs, ARBs)

**FIGURE 6:**
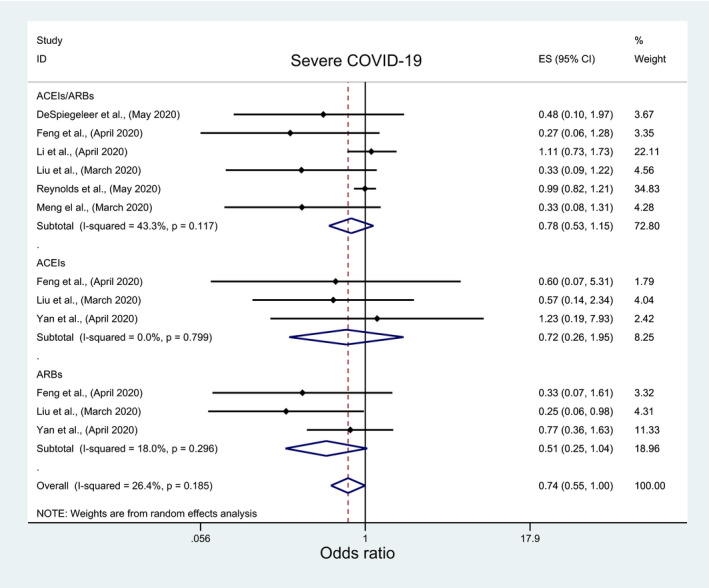
Forest plot depicting pooled estimates for the association between developing severe COVID‐19 infection and the three levels of renin‐angiotensin system drug exposure (ACEIs/ARBs, ACEIs, ARBs)

The overall pooled analysis of nine studies indicated insignificant association between the risk of acquiring COVID‐19 infection and the use of ACEIs/ARBs (OR: 1.01; 95%CI: 0.93, 1.10), ACEIs (OR: 1.13; 95%CI: 0.9, 1.42), or ARBs (OR: 0.56; 95%CI: 0.11, 2.89) (Figure 5; Table 3). The subgroup analyses results were consistent with overall analyses results for ACEIs/ARBs and ACEIs (File S5A and B; Table 4) but they were inconsistent for ARBs with a significantly lower risk of acquiring COVID‐19 with ARBs among nonpeer‐reviewed studies, good‐quality studies and studies which reported crude measure of effects (OR: 0.24; 95%CI: 0.17, 0.34) (File S5C; Table 4). Similarly, in a pooled analysis of seven and two studies, insignificant association was observed between the risk of developing severe COVID‐19 infection, severe pneumonia, respectively, and ACEIs/ARBs (OR:0.78; 95%CI: 0.53, 1.15; OR:1.29; 95%CI: 0.24, 6.96), ACEIs (OR: 0.72; 95%CI: 0.26, 1.95) or ARBs (OR: 0.51; 95%CI: 0.25, 1.04) (Figure 6; Table 3), regardless of any subgroup analysis (File S6; Table 4).

#### 3.2.3 Hospitalization, hospital discharge and duration of hospital stay

**FIGURE 7:**
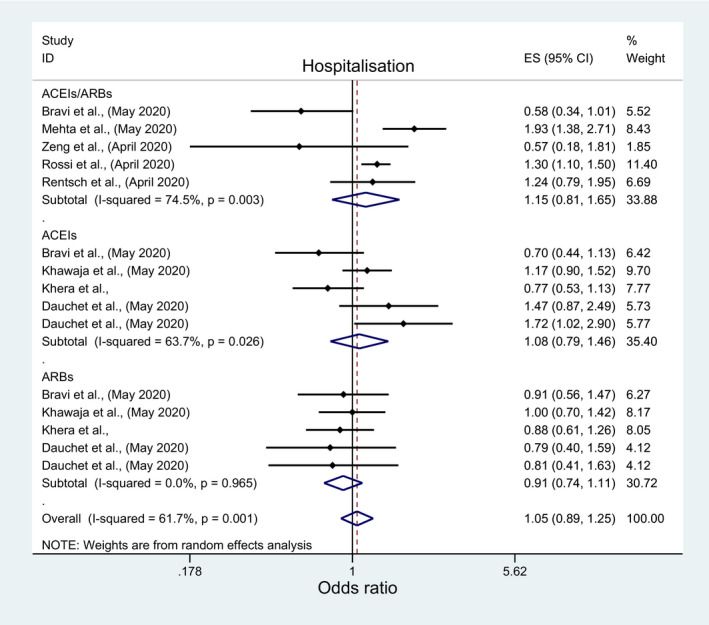
Forest plot depicting pooled estimates for the association between hospitalization and the three levels of renin‐angiotensin system drug exposure (ACEIs/ARBs, ACEIs, ARBs)

**FIGURE 8:**
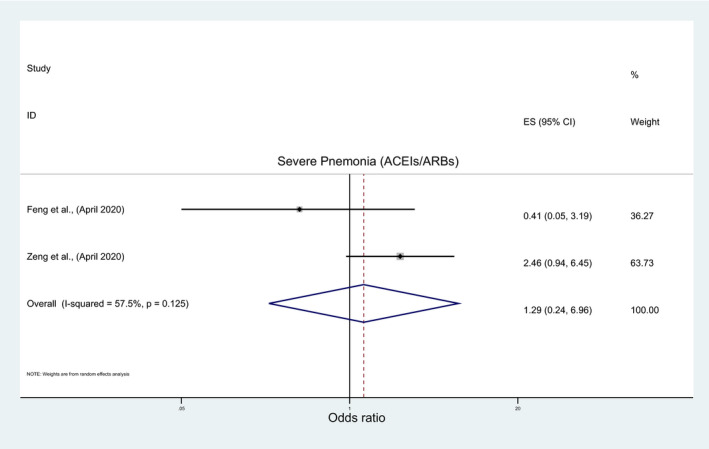
Forest plot depicting pooled estimate for the association between hospital discharge and ACEIs/ARBs use

In a pooled analysis of eight and three studies, there was no signification association between hospitalization, hospital discharge rate and ACEIs/ARBs (OR: 1.15; 95%CI: 0.81, 1.65; OR: 1.21; 95%CI: 0.74, 1.99), ACEIs (OR: 1.08; 95%CI: 0.79, 1.46) or ARBs (OR: 0.91; 95%CI: 0.74, 1.11) (Figure 7; Figure 8 and Table 3). However, subgroup analyses demonstrated a significantly higher risk of hospitalization with ACEIs/ARBs among studies conducted in the USA (OR:1.59; 95%CI: 1.03, 2.44), peer‐reviewed studies (OR:1.93, 95%CI: 1.38, 2.71), good quality studies and studies which reported adjusted measure of effect (OR:1.30, 95%CI: 1.10, 1.50) (File S7; Table 4). Contrastingly, a significantly higher rate of hospital discharge was observed with ACEIs/ARBs but only among nonpeer‐reviewed articles (OR:1.51; 95%CI: 1.18, 1.93) (File S8; Table 4). Two studies reported data on the duration of hospital stay. Both were in favor of ACEIs/ARBs with Yang et al^25^ reporting a significant reduction in the mean duration of hospital stay of 2.3 days (95%CI: −3.61, −0.99) with ACEIs/ARBs while Zeng et al^26^ reported a lower median duration of hospital stay of 21 days (IRQ: 15‐25) with ACEIs/ARBs versus 22 days (IQR: 16‐28) with non‐ACEI/ARB use.

#### 3.2.4 Use of a ventilator

**FIGURE 9:**
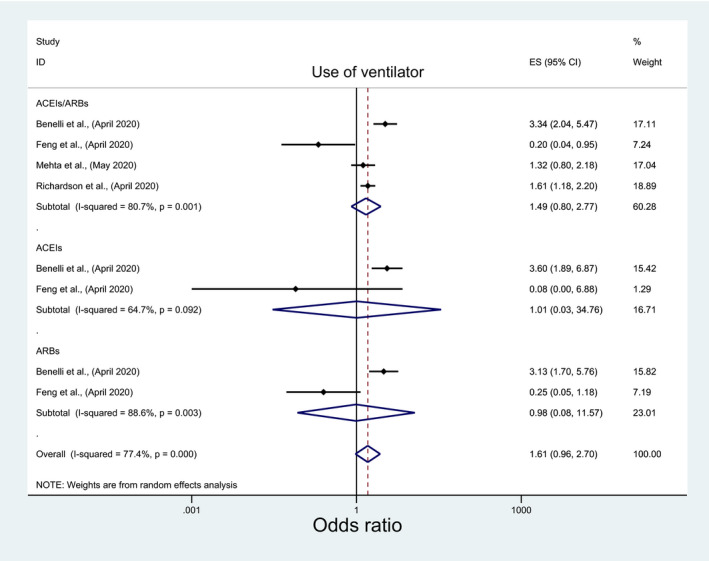
Forest plot depicting pooled estimates for the association between use of ventilator and the three levels of renin‐angiotensin system drug exposure (ACEIs/ARBs, ACEIs, ARBs)

**FIGURE 10:**
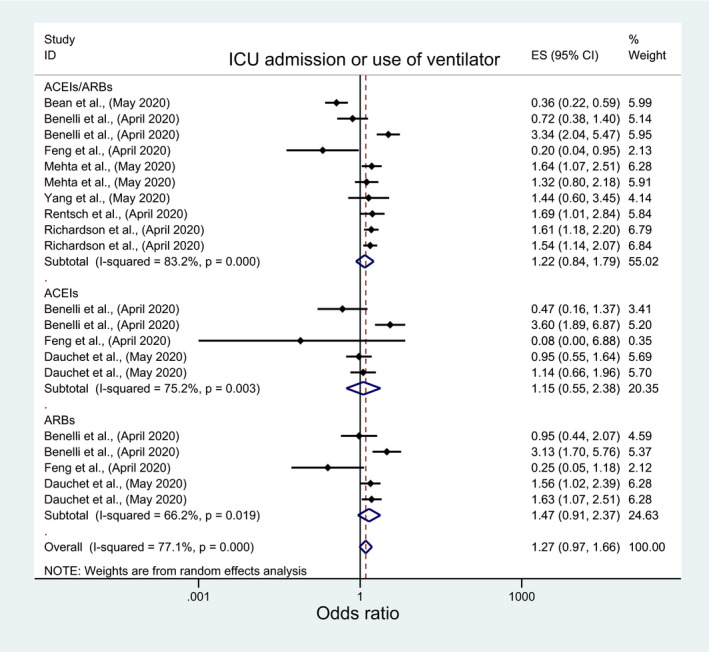
Forest plot depicting pooled estimates for the association between use of ventilator/Intensive Care Unit admission and the three levels of renin‐angiotensin system drug exposure (ACEIs/ARBs, ACEIs, ARBs)

Among pertinent studies, there was no significant association between these outcomes and the use of ACEIs/ARBs (OR:1.49; 95%CI: 0.80, 2.77; OR: 1.26; 95%CI: 0.84, 1.80), ACEIs (OR:1.01; 95%CI:0.03, 34.76; OR:1.15; 95%: 0.55, 2.38), or ARBs (OR:0.98; 95%CI: 0.08, 11.57; OR: 1.48; 95%CI: 0.91, 2.38) (Figures 9 and 10; Table 3). However, significantly higher odds of ventilator use with ACEIs/ARBs among the European studies (OR: 3.34; 95%CI: 2.04, 5.48) and the USA (OR:1.52; 95%CI:1.17, 1.98) in contrast to significantly lower odds among those from Asia (OR:0.2; 95%CI: 0.04, 0.95) (File S9, Table 4). Contrastingly, significantly higher odds of ventilator use with ACEIs/ARBs was only observed among nonpeer‐reviewed studies (OR:3.34; 95%CI: 2.04, 5.48) (File S9, Table 1).

#### 3.2.5 Other miscellaneous outcomes

Zhang et al^21^ reported a significantly lower rate of septic shock (HR: 0.32; 95%CI: 0.13, 0.8) as well as nonsignificant lower rate of ARDS (HR: 0.65; 95%CI: 0.41, 1.04), acute kidney injury (HR:0.78; 95%CI: 0.37, 1.65), and cardiac injury (HR: 0.76; 95%CI: 0.44, 1.32) among ACEI/ARB users. Furthermore, Richardson et al,^24^ reported lower odds of hospital readmission with ACEIs/ARBs (OR: 0.77; 95%CI: 0.30, 1.94), albeit nonsignificant.

### 3.3 Publication bias

Results from the funnel plot (File [Link], [Link]) and Egger's asymmetry test for the death outcome, which was the only outcome whereby >10 studies were included in the meta‐analysis, indicated statistically insignificant evidence of publication bias (bias coefficient:0.85, 95%CI: −2.23, 3.93, *P* = .445).

## 4 DISCUSSION

The pooled analyses in this updated systematic review and meta‐analysis indicated no evidence of any significant association between ACEIs/ARBs and any COVID‐19 related clinical outcomes; however, the subgroup analyses revealed evidence of a negative impact of ACEIs/ARBs use and some COVID‐19‐related clinical outcomes such as higher odds of hospitalization, ICU admission and ventilator use. Contrastingly, a positive impact were observed in terms of lower odds of death/ICU admission, as a composite outcome, and a higher rate of hospital discharge. Furthermore, our study findings, for the first time, showed interclass variations between ACEIs and ARBs effects on COVID‐19 clinical outcomes with low‐quality evidence indicating lower risk of acquiring COVID‐19, less severe COVID‐19 infection, higher rate of ICU admission and ventilator use with ARBs but not ACEIs.

Our study findings also showed no significant association between ACEIs/ARBs and mortality, severe COVID‐19 infection, or positive tests for COVID‐19, in agreement with two previously published systematic reviews.^29^, ^30^ This was despite the inclusion of more recently published studies,^18^, ^27^, ^40^, ^41^, ^49^, ^50^, ^53^ which implies consistency of evidence. This is encouraging given the controversies surrounding hydroxychloroquine. Furthermore, these nonsignificant associations were also observed for additional COVID‐19‐related outcomes including ICU admission, hospitalization, and hospital discharge. However, unlike the previous two systematic reviews,^29^, ^30^ our study found evidence of associations between ACEI/ARB use and certain COVID‐19 clinical outcomes. While the pooled estimate of the subgroup analyses indicated a higher odds of ICU admission with ACEIs/ARBs among studies conducted in the USA^23^, ^43^, ^44^ and peer‐reviewed studies,^23^, ^25^, ^44^ all these studies were of poor quality and none performed adjusted analyses to account for potential confounders. Confounding by indication is of particular concern with comorbidities such as CVD and diabetes associated with more severe COVID‐19 morbidity and mortality.^4^, ^5^, ^6^ Similarly, the observed significant associations between ACEIs/ARBs use and high odds of ventilator use and hospital discharge rates were from Benelli et al^41^ and Ip et al^27^ and Zeng et al,^26^ respectively, all of which were nonpeer‐reviewed, of poor quality and used crude analyses. Similarly, the studies in the pooled analyses that showed significant association of ARBs use and ICU admission,^41^, ^42^ lower risk of acquiring COVID‐19 infection,^48^ and severe infection^18^, ^19^ were of poor quality, used unadjusted/crude analyses, and/or nonpeer‐reviewed. In terms of duration of hospital stay, Yang et al^25^ and Zeng et al^26^ both reported a reduction in hospital stay with ACEIs/ARBs; however, it was not possible to combine them in the meta‐analysis as they used a different measure of effects with the former reporting the outcome as a mean difference with the latter as a median.

On the other hand, our study findings showed some high‐quality evidence on the association of ACEIs/ARBs and higher odds of hospitalization but lower odds of death/ICU admission (as a composite endpoint). A higher odd of hospitalization was observed in the subgroup analyses of studies conducted in the USA^43^, ^44^ although it should be noted that there was some heterogeneity (57.7%) between the USA studies, used adjusted analyses,^47^ peer‐reviewed^44^ and of good quality^47^; whereas the studies for lower death/ICU admission were from Europe,^40^, ^45^ used adjusted analyses and of good quality,^40^ although none of them were peer‐reviewed.

Several hypotheses have been suggested to explain the negative and positive effects of ACEIs/ARBs use on COVID‐19 clinical outcomes. The former is thought to be related to ACEIs/ARBs potential ability to upregulate ACE2, the cell entry point for COVID‐19; hence facilitate COVID‐19 cell entry and its subsequent infectivity/pathogenicity^55^; however, the evidence to date demonstrates ACE2’s upregulation consistently in cardiac and renal tissues in response to ARBs therapy but not ACEIs^4^, ^56^; this observed difference between ARBs and ACEIs has been suggested to be due to the increased level of angiotensin II, which occurs following ARBs treatment but not ACEIs, which in turn imposes an increased substrate load on ACE2 enzyme requiring its upregulation.^57^ Importantly, it should be emphasised that evidence of ACEIs/ARBs induced ACE2 upregulation in the respiratory tracts, which is the key entry system for COVID‐19, is lacking.^56^ Furthermore, it should be noticed that alteration in angiotensin II level, which is only one substrate of ACE2’s multiple substrates, is unlikely to result in any meaningful differences in ACE2 substrate load, hence its upregulation^56^; additionally, the fact that people from various sexes, ages, and races are all susceptible to COVID‐19 infection suggests that physiological expression of ACE2 might already be sufficient for COVID‐19 infection; thus any further ACE2 upregulation might not have effects on the risk/severity of COVID‐19 infection.^25^ Together, these evidences indicate that the concerns around ACEIs/ARBs use in COVID‐19 patients might be unjustifiable. On the other hand, the protective effect hypothesizes on ACEIs/ARBs protecting against lung injury, through blockage of the harmful angiotensin II‐ AT1R axis, which gets activated by impairment of ACE2 activity as a result of ACE2’s downregulation results from ACE2’s binding with COVID‐19 virus; additionally, the corresponding increase in angiotensin II and angiotensin I, due to ACEIs/ARBs use, would activate the protective axis and hence reduce COVID‐19 viral pathogenicity.^4^ Genetic ACE2 polymorphism among some individuals has been also suggested as potential factor explaining, at least partially, the harmful effects on ACEIs/ARBs among COVId‐19 patients^58^; but this needs further investigation.

### 4.1 Strengths and limitation

We believe this study is the first to provide a systematic, comprehensive and updated evaluation of the effects of ACEIs/ARBs on all the reported COVID‐19‐related clinical outcomes including exploration of interclass differences between ACEIs and ARBs as well as multiple subgroup analyses, although we do acknowledge that some of the subgroup analyses only had 1‐2 studies for some of the studied outcomes such as ICU admission and Death/ICU admission. However, our study has limitations. Since all included studies were observational studies, the effect of confounding including residual confounders cannot be ruled out. There is also the possibility that new studies have been published since our review. However, we included nonpeer‐reviewed articles published in medRxiv to help address this.

## 5 CONCLUSION

There appears to be no evidence of association between ACEIs/ARBs use and a wide range of COVID‐19‐related clinical outcomes. However, good quality evidence exists for ACEIs/ARBs and higher odds of hospitalization, lower odds of death/ICU admission (as composite endpoint); but only low‐quality evidence for higher ICU admission, ventilator use, hospital discharge and lower duration of hospital stay exists. Furthermore, there is evidence, albeit of poor quality, of differences between ACEIs and ARBs with the latter being associated with significantly higher ICU admission but lower COVID‐19 infection risk and severity. Given the continuing controversial and paradoxical clinical studies’ findings and hypotheses, we believe it is necessary to continue to evaluate the effects of ACEIs/ARBs on COVID‐19 clinical outcomes especially as more randomized studies are reported.

## 6 NOMENCLATURE OF TARGETS AND LIGANDS

Key protein targets and ligands in this article are hyperlinked to corresponding entries in http://www.guidetopharmacology.org, the common portal for data from the IUPHAR/BPS Guide to PHARMACOLOGY,^59^ and are permanently archived in the Concise Guide to PHARMACOLOGY 2019/20.^60^

## ETHICS APPROVAL

Not required.

## DISCLOSURE

Nothing to declare.

## AUTHOR CONTRIBUTIONS

Study conception and design: all authors; data collection and management: NA, AL; data analysis and interpretation: AK, BG; manuscript writing and drafting: AK, NA; manuscript reviewing and revising as well as providing constructive criticism and final approval: all authors.

## Supporting information

File S1

File S2

File S3

File S4

File S5A

File S5B

File S5C

File S6

File S7

File S8

File S9

File S10

## Data Availability

The data that support the findings of this study are available from the corresponding author upon reasonable request.
