# Supplementary material for: A systematic review and meta‐analysis of the use of renin‐angiotensin system drugs and COVID‐19 clinical outcomes: What is the evidence so far?
Source: Pharmacol Res Perspect. 2020 Oct 20;8(6):e00666. doi: 10.1002/prp2.666 (PMC7575889; doi:10.1002/prp2.666)
Supplement: Supplementary file 1 — File S1 [file PRP2-8-e00666-s001.docx]

| **Search terms** | **Results** |
| --- | --- |
| **Medline (OVID)** | |
| (“renin-angiotensin system”.mp. OR Angiotensin Receptor Antagonists/ OR “Angiotensin II receptor Antagonists”. mp. OR Angiotensin-Converting Enzyme Inhibitors/) AND (Coronavirus/ OR coronavirus infections/ OR COVID19.mp. OR “COVID-19”.mp. OR “corona-virus”.mp. OR “severe acute respiratory syndrome coronavirus 2”.mp.) | 43 |
| **Embase** | |
| (“renin-angiotensin system”.mp. OR angiotensin Receptor Antagonists/ OR “Angiotensin converting Enzyme Inhibitors”.mp.) AND (“Coronavirus”.mp. OR Coronavirus infection/ OR “COVID-19”.mp. OR COVID19.mp. OR “corona-virus”.mp. OR “severe acute respiratory syndrome coronavirus 2”.mp.) | 115 |
| **Scopus** | |
| ((TITLE-ABS-KEY(Coronavirus)) OR (TITLE-ABS-KEY(COVID19)) OR (TITLE-ABS-KEY(corona-virus)) OR (TITLE-ABS-KEY(COVID-19)) OR (TITLE-ABS-KEY("severe acute respiratory syndrome coronavirus 2"))) AND ((TITLE-ABS-KEY("renin--angiotensin system")) OR (TITLE-ABS-KEY("Angiotensin Receptor Antagonists")) OR (TITLE-ABS-KEY("Angiotensin II Receptor Antagonists")) OR (TITLE-ABS-KEY("Angiotensin Converting Enzyme Inhibitors"))) | 156 |
| **MedRixiv** | |
| (Coronavirus OR COVID-19 OR “severe acute respiratory syndrome coronavirus 2” OR corona-virus) AND (“renin–angiotensin system” OR “Angiotensin Receptor Antagonists” OR “Angiotensin II Receptor Antagonists” OR ACEIs OR ARBs OR “Angiotensin Converting Enzyme Inhibitors”) | 108 |
