# Supplementary figures and images for: A systematic review and meta‐analysis of the use of renin‐angiotensin system drugs and COVID‐19 clinical outcomes: What is the evidence so far?
Source: Pharmacol Res Perspect. 2020 Oct 20;8(6):e00666. doi: 10.1002/prp2.666 (PMC7575889; doi:10.1002/prp2.666)

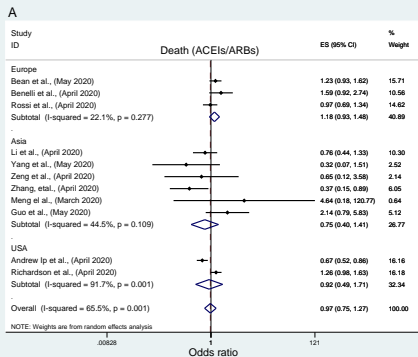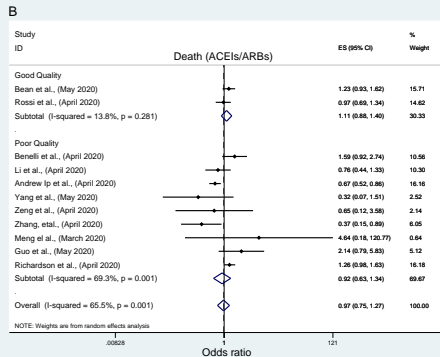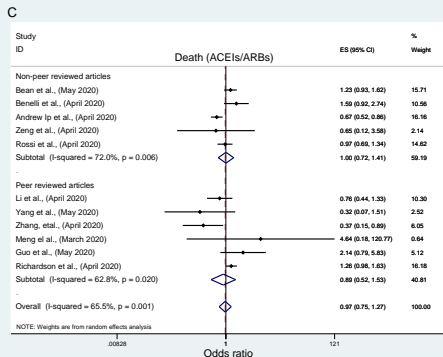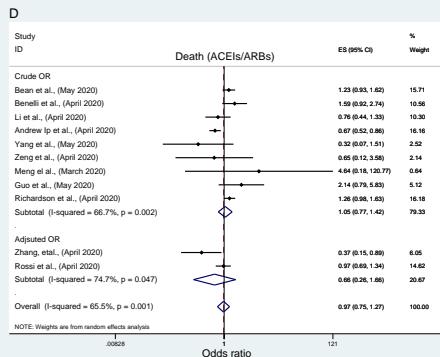

Supplement: Supplementary file 2 — File S2 [file PRP2-8-e00666-s002.pdf]

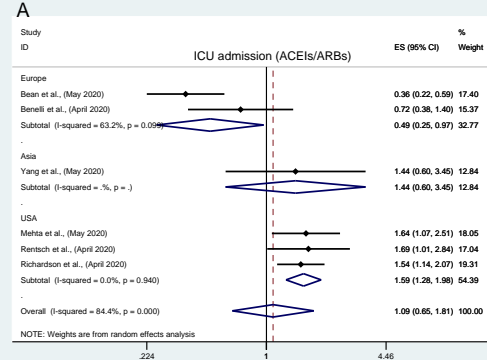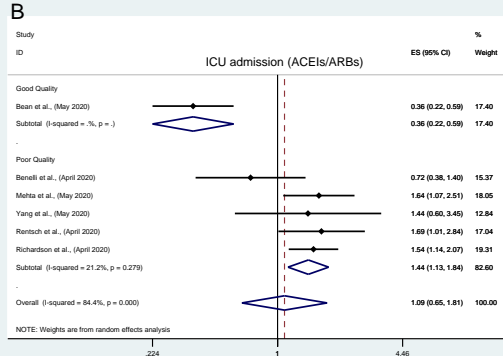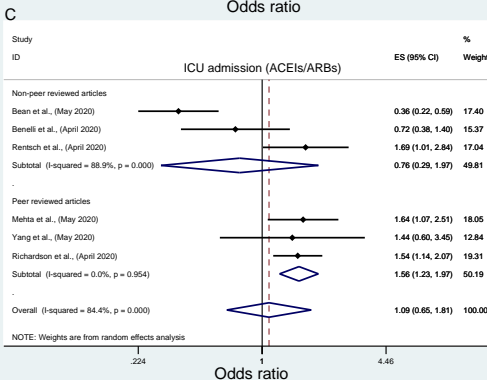

Supplement: Supplementary file 3 — File S3 [file PRP2-8-e00666-s003.pdf]

A

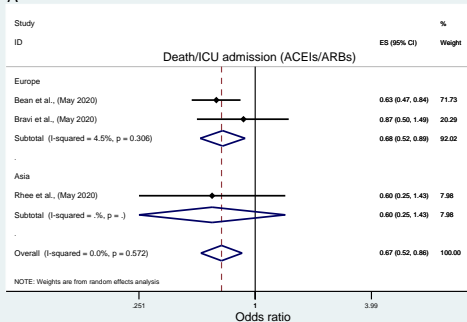

B

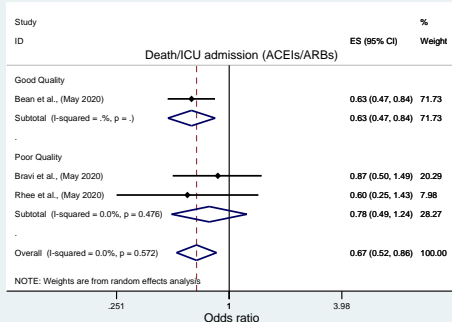

C

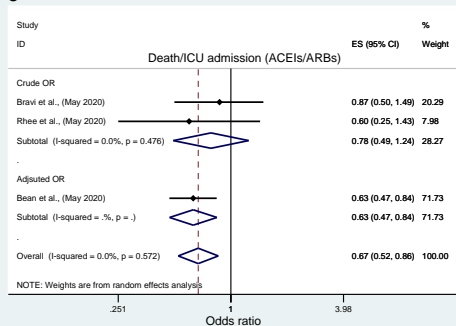

Supplement: Supplementary file 4 — File S4 [file PRP2-8-e00666-s004.pdf]

A

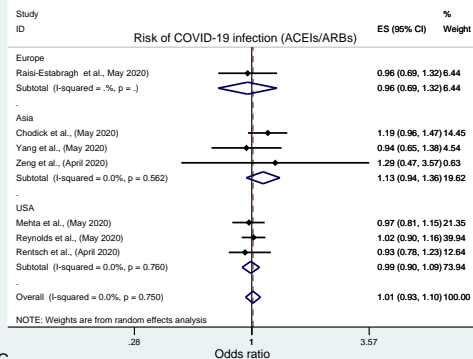

B

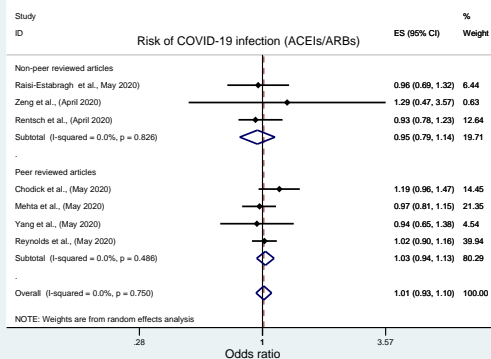

C

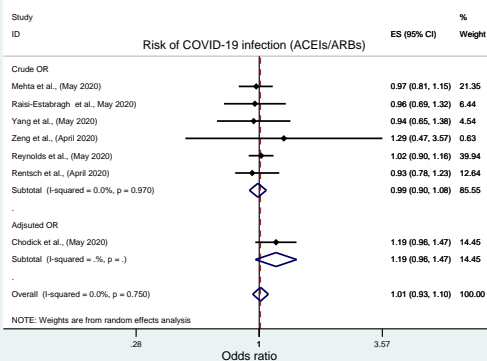

Supplement: Supplementary file 5 — File S5A [file PRP2-8-e00666-s005.pdf]

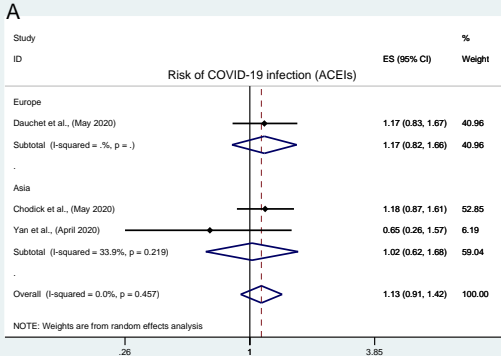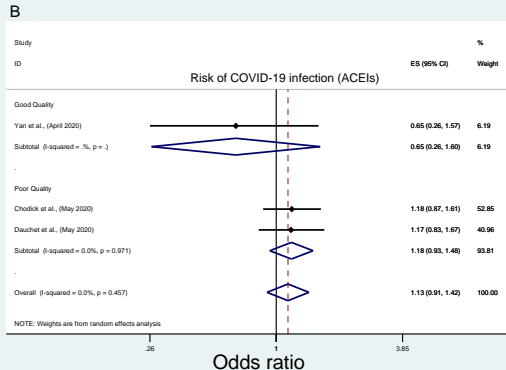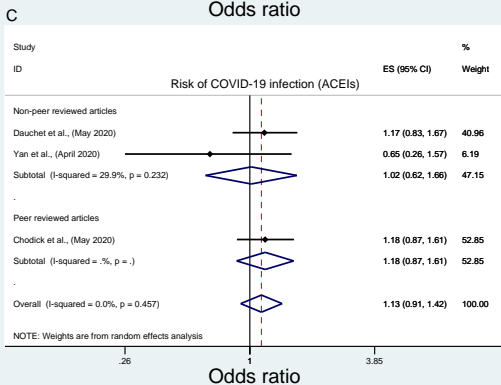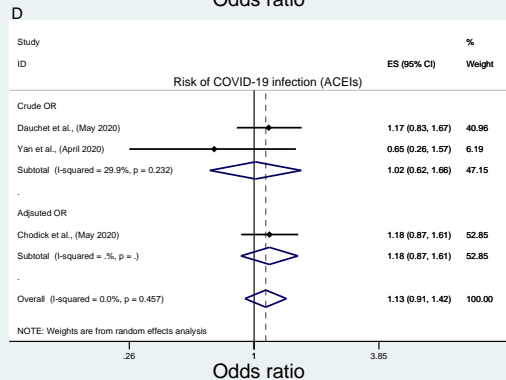

Supplement: Supplementary file 6 — File S5B [file PRP2-8-e00666-s006.pdf]

A

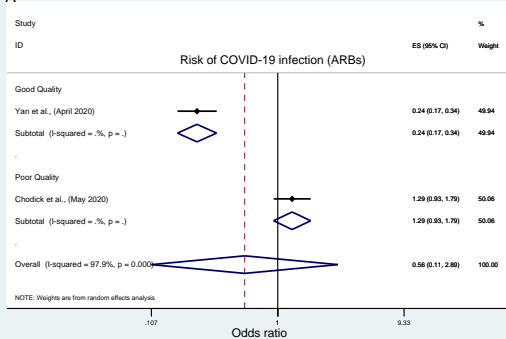

B

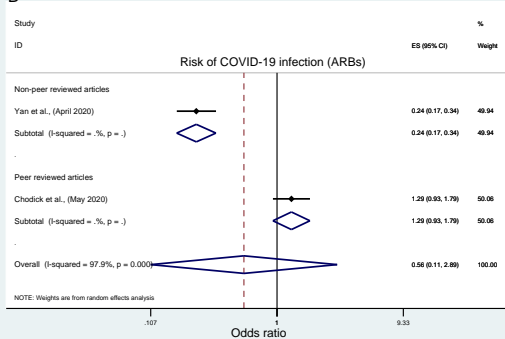

C

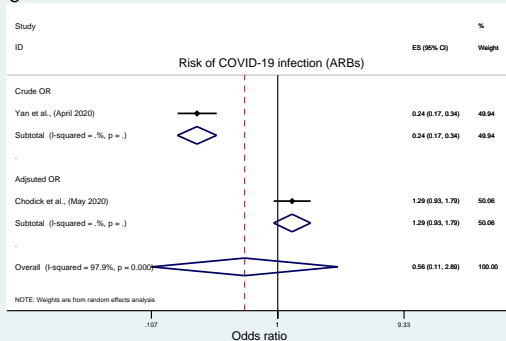

Supplement: Supplementary file 7 — File S5C [file PRP2-8-e00666-s007.pdf]

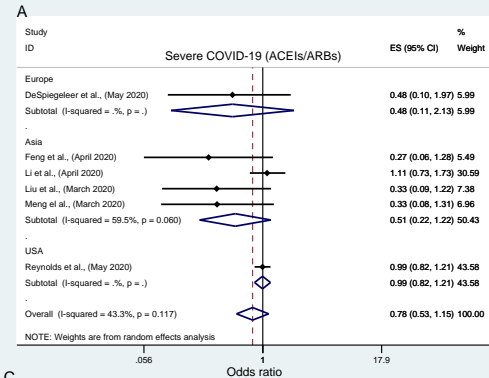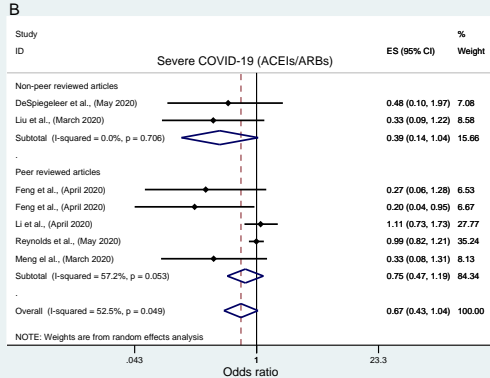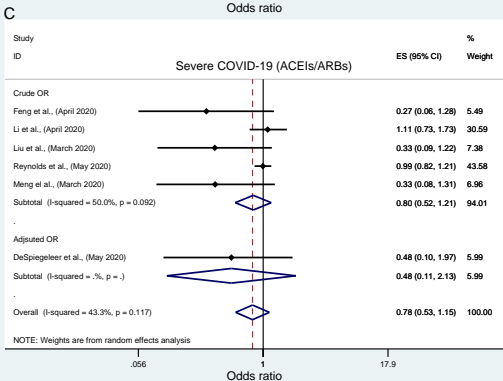

Supplement: Supplementary file 8 — File S6 [file PRP2-8-e00666-s008.pdf]

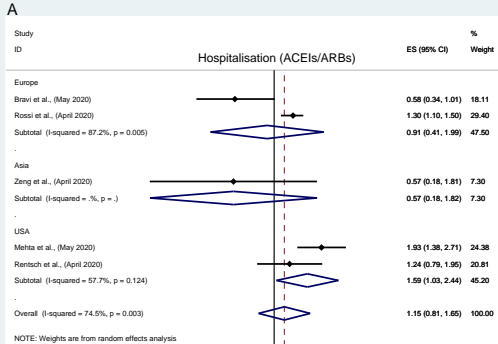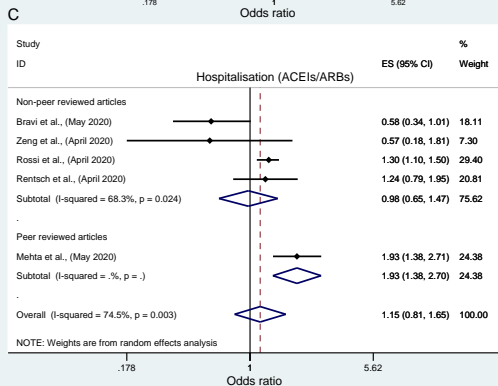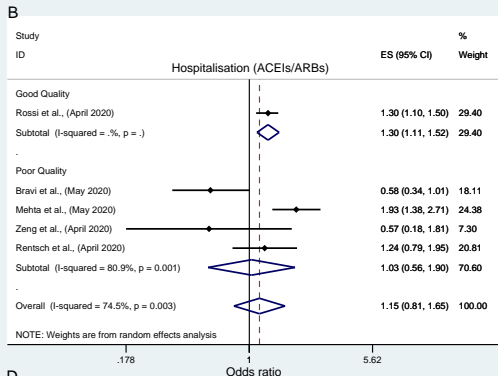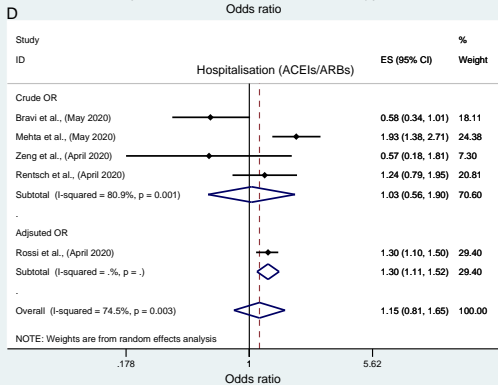

Supplement: Supplementary file 9 — File S7 [file PRP2-8-e00666-s009.pdf]

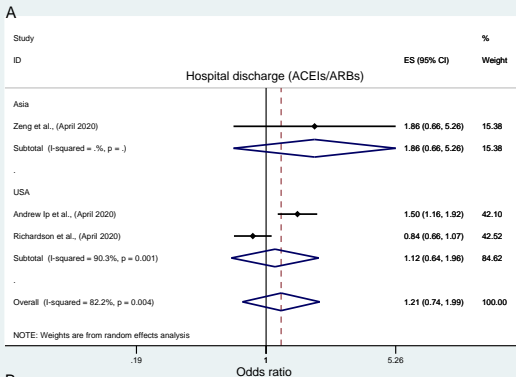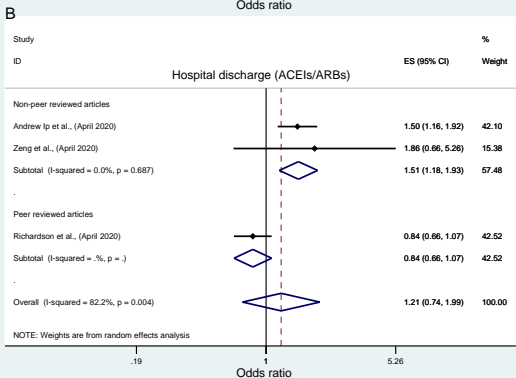

Supplement: Supplementary file 10 — File S8 [file PRP2-8-e00666-s010.pdf]

A

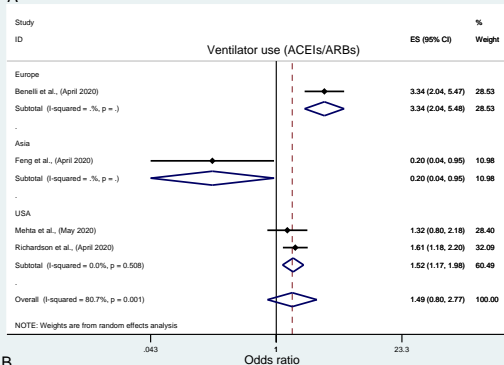

B

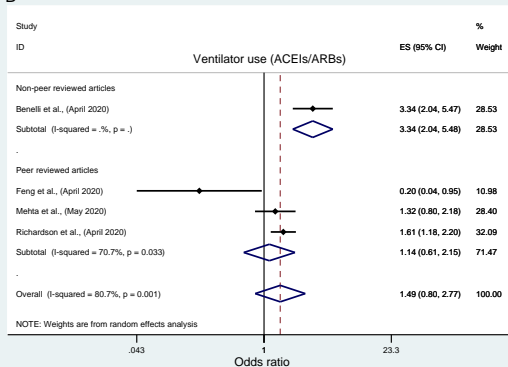

Supplement: Supplementary file 11 — File S9 [file PRP2-8-e00666-s011.pdf]

Standard error of log OR

Log OR

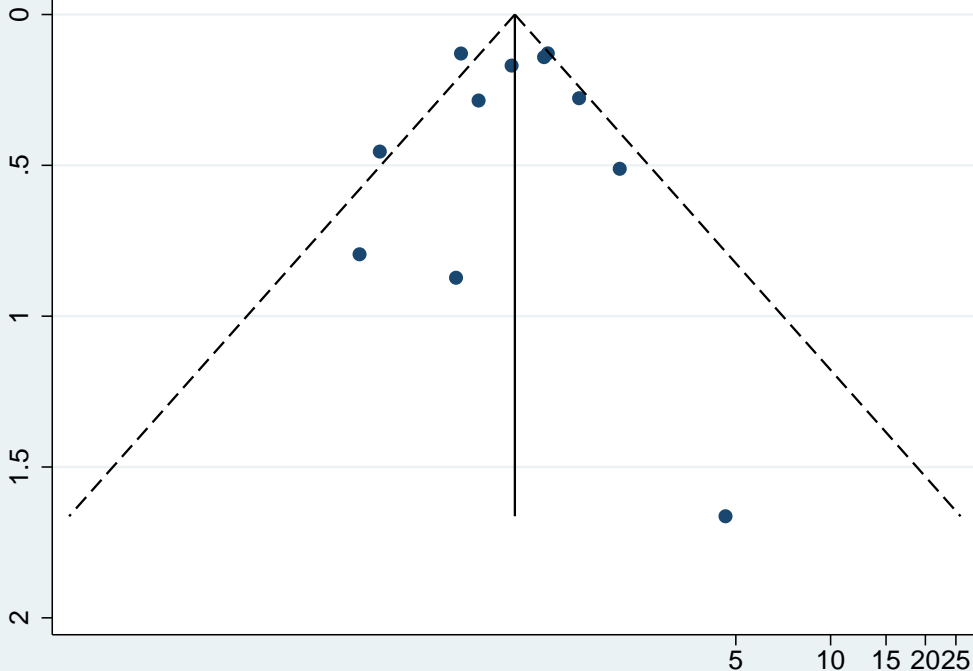

Supplement: Supplementary file 12 — File S10 [file PRP2-8-e00666-s012.pdf]
